# Legacy, meaning-making, and biographical music therapy in palliative and end-of-life care: a narrative review

**DOI:** 10.3389/fpsyg.2026.1913381

**Published:** 2026-10-20

**Authors:** Jared Kyle Sieusahai

**Affiliations:** Faculty of Medicine and Dentistry, University of Alberta, Edmonton, AB, Canada

**Keywords:** biographical music therapy, end-of-life care, legacy, meaning-making, music therapy, palliative care, songwriting, spiritual wellbeing

## Abstract

**Background/objectives:**

Legacy work and meaning-making address existential, spiritual, and relational needs in palliative and end-of-life care. Music therapy offers opportunities for biographical reflection, emotional expression, and the creation of personally meaningful recordings. This narrative review examines its clinical applications, reported outcomes, proposed processes, and limitations, while distinguishing legacy-focused interventions from related supportive practices.

**Methods:**

A structured literature search was undertaken through PubMed and the University of Alberta Library Primo discovery platform in June 2026, with targeted source verification and supplementary searches during revision in September 2026. Published trials, qualitative and mixed-methods research, reviews, and clinical accounts were synthesized narratively. Study design, population, comparator, music selection, and limitations were considered separately; no formal risk-of-bias grading or pooled analysis was undertaken.

**Results:**

The Song of Life randomized trial supports short-term benefits in spiritual wellbeing and ego-integrity, but did not demonstrate improvement in its primary psychological quality-of-life outcome. Other randomized trials address relaxation, pain, anxiety, or caregiver outcomes and should not be interpreted as independent confirmation of legacy-specific effects. Qualitative research describes opportunities for expression, connection, and remembrance, whereas sustained bereavement benefits remain uncertain. Studies generally use individualized music or describe intervention methods rather than compare genres directly. Research from Singapore adds culturally situated evidence, but generalizability across populations remains limited. Evidence concerning opioid use is mixed and setting-specific: a recent inpatient cohort found no significant exposure-by-time effect on pain or opioid utilization. Music thanatology, neonatal heartbeat recordings, and music therapy surrounding medical assistance in dying provide related but distinct evidence.

**Conclusion:**

Biographical and legacy-oriented music therapy may complement multidisciplinary palliative care, particularly when aligned with patients’ preferences and goals. The evidence supports differentiated claims about short-term outcomes and perceived meaning, rather than a universal mechanism or established long-term benefit. Priorities include independent replication, culturally grounded research, clearer reporting of musical content, and longitudinal assessment of family outcomes.

## 1 Introduction

The existential dimensions of dying, including confronting mortality, integrating a life’s meaning, preserving identity beyond illness, and maintaining relational connection, are important concerns in palliative and oncology care. Palliative care addresses physical, psychological, social, and spiritual needs, and meaning-centred psychotherapy provides one example of a structured approach to existential distress (Saunders, 2000; Breitbart et al., 2010). Music therapy can complement this work by offering musical as well as verbal opportunities for communication and reflection (Schmid et al., 2018). Its contribution should not be framed as replacing symptom management or as uniquely meeting needs that other psychosocial approaches cannot address.

Music therapy occupies a distinctive position in this landscape because musical engagement can involve autobiographical associations, emotional experience, and interpersonal communication (Koelsch, 2014; Schmid et al., 2018). Legacy-oriented and biographical approaches extend these possibilities through personally meaningful song selection, songwriting, musical life review, and recordings intended for patients or families (O’Callaghan, 1996; Heath and Lings, 2012; Valenti et al., 2026). A song created with a therapist may become a keepsake, but the value of the process and the subsequent use of the recording are separate outcomes requiring separate evaluation.

This review synthesizes evidence on legacy-oriented and biographical music therapy in palliative and end-of-life care. Songwriting and musical life review constitute its central focus. Caregiver interventions, pediatric and neonatal applications, music thanatology, and music therapy surrounding medical assistance in dying (MAiD) are considered where they illuminate related questions of connection, remembrance, or accompaniment. They differ in their theoretical foundations, intended outcomes, populations, and evidence bases, and are not treated as interchangeable forms of a single intervention.

The multicentre Song of Life (SOL) trial provides directly relevant randomized evidence for a structured biographical intervention (Warth et al., 2021, 2019). Alongside this, a growing body of qualitative and mixed-methods literature explores experiences of music therapy, legacy creation, and family connection (Schmid et al., 2018; Gillespie et al., 2024; Valenti et al., 2026). This review asks what these approaches involve, which outcomes their evidence supports, how music selection and cultural context may shape delivery, and where claims about effectiveness or proposed mechanisms remain uncertain.

## 2 Methods

This narrative review uses the Scale for the Assessment of Narrative Review Articles (SANRA) as a guide to transparent reporting and critical reasoning (Baethge et al., 2019). SANRA is an appraisal scale, not a systematic-review reporting guideline. A narrative approach was chosen to integrate clinical, experiential, and conceptual literature around legacy and meaning-making, rather than estimate a pooled treatment effect. Heterogeneous designs do not themselves preclude systematic review; the present work does not claim an exhaustive or reproducible systematic-review process (Grant and Booth, 2009).

Terminology is used specifically. Legacy-oriented music therapy describes work explicitly directed toward creating or sharing something of personal significance beyond the immediate encounter. Biographical music therapy refers to structured engagement with a patient’s life narrative through meaningful music, exemplified by SOL. Songwriting involves collaborative creation of a song, while musical life review uses music to support reflection on life experiences. Music thanatology is a related bedside practice, commonly involving harp and voice, oriented toward accompaniment and comfort rather than necessarily toward biography or artifact creation (Warth et al., 2019; Heath and Lings, 2012; Schroeder-Sheker, 1994; Freeman et al., 2006). These distinctions define the limits of the synthesis.

### 2.1 Search strategy

The original search was conducted through PubMed and the University of Alberta Library Primo discovery platform in June 2026. Primo was used as a discovery interface; searching it is not equivalent to executing separate database-specific searches in PsycINFO, CINAHL, MEDLINE, Web of Science, or Scopus. The original search record reported the following term pairs and returned-record counts: biographical music therapy AND palliative care (Koelsch, 2014); legacy music therapy AND end of life (Giordano et al., 2022); meaning making music therapy AND palliative (Heath and Lings, 2012); songwriting music therapy AND palliative cancer (Warth et al., 2019); music therapy existential AND dying patients (Heath and Lings, 2012); and life review music therapy AND terminal illness (Black et al., 2020). Supplementary searches were Song of Life music therapy AND palliative (88); music therapy dignity AND end of life (Choi, 2010); and music thanatology AND palliative care (Grant et al., 2009). These are platform search returns, not deduplicated inclusion counts. PubMed searches and reference-list checking supplemented this process.

During revision in September 2026, targeted searches of publication titles and combinations of music therapy with genre, culture, palliative care, MAiD, heartbeat recordings, pain, and opioid utilization were used to verify cited reports and address reviewer questions. PubMed/Europe PMC records and accessible publisher reports were consulted. This update was targeted rather than a rerun of all original searches. English-language retrieval and the absence of complete database-specific search histories limit reproducibility and coverage.

### 2.2 Inclusion and exclusion criteria

The central literature concerned music therapy, biographical interventions, songwriting, life review, and legacy work in palliative, hospice, oncology, or end-of-life settings, published in English from 1990 through the September 2026 revision. Trials, observational studies, qualitative and mixed-methods studies, and evidence syntheses were eligible. Clinical and theoretical accounts were used to explain practice and concepts, not to establish efficacy. Foundational background sources could precede 1990. Conference abstracts alone were not used to support treatment-effect claims.

Related oncology symptom-management trials, neonatal legacy studies, and music-thanatology reports were retained as contextual evidence and identified as such. In particular, treatment during chemotherapy or transplantation is not assumed to represent end-of-life care, and neonatal intensive care is not synonymous with neonatal palliative care. General palliative-care cultural studies inform contextual considerations but do not demonstrate the effectiveness of music therapy.

### 2.3 Screening, appraisal, and synthesis

The author screened and selected the original literature for relevance. The synthesis distinguishes evidence directly addressing biographical or legacy work from related symptom-management and contextual literature. The tables summarize key evidence discussed in the narrative rather than constitute a systematic-review inclusion inventory.

No validated study-level risk-of-bias instrument was applied. Appraisal is descriptive and considers study design, comparator, reporting, and applicability rather than assigning numerical or high-to-low grades. A qualitative study is not intrinsically low quality because it cannot estimate a treatment effect, and a systematic review does not make its underlying evidence high certainty. Three tables distinguish randomized evidence, nonrandomized and qualitative research, and evidence syntheses or clinical accounts. Entries are chronological within each table. Reports from the same SOL trial are identified as linked publications rather than independent replications. The proposed processes in Section 3.2 are interpretive themes, not a validated mechanistic model.

## 3 Results

Table 1 summarizes randomized evidence and the linked SOL substudy; Table 2 presents selected nonrandomized and qualitative research; Table 3 summarizes evidence syntheses and descriptive clinical literature. The tables identify musical content or selection procedures where available. Intervention format, instrumentation, musical genre, and personal preference are recorded as different characteristics. Absence of a genre description is not evidence that genre was irrelevant.

**Table 1:** Randomized studies and the linked SOL analysis.

Study and setting	Design and population	Intervention and music selection/genre	Comparator and principal finding	Interpretive limits
Choi (2010)	Four-arm randomized study; 32 hospice family caregivers	Music, PMR, or their combination; genre NR-A	Control group; no significant main effect of group	Small sample; caregiver outcomes, not patient or legacy outcomes; time effects do not establish music-specific benefit
Gutgsell et al. (2013), United States	Randomized trial; 200 palliative-care inpatients	Single session of therapist-guided autogenic relaxation with live music; genre NR-A	Standard care including analgesics; greater immediate reduction in numerical pain scores	Usual-care comparator; short-term endpoint; not a legacy intervention or demonstration of opioid reduction
Warth et al. (2015), Germany	Randomized trial; 84 palliative-care inpatients	Two live relaxation sessions using monochord and voice; defined delivery rather than a genre comparison	Recorded verbal relaxation; relaxation and wellbeing improved, acute pain difference not significant	Unblinded intervention; short follow-up; no explicit legacy component
Bates et al. (2017), United States	Randomized study; 82 autologous stem-cell transplant patients	Two interactive sessions with a board-certified music therapist; genre NR-A	No MT; similar pain scores, lower morphine-equivalent use with MT (median 24 vs. 73 mg; *p* = 0.038)	Medication use was a secondary outcome; transplant setting, not an end-of-life cohort
Warth et al. (2021, 2019), Germany	Multicentre trial; 104 palliative-care patients, 52 per arm	Three-session SOL protocol; personally meaningful song, live rendition and recording; individualized repertoire	Relaxation exercises; spiritual wellbeing and ego-integrity favoured SOL; primary psychological QoL and global QoL did not differ significantly	Short follow-up, no participant blinding; outcome-specific benefit; limited cultural transferability
Koehler et al. (2022), Germany	Linked SOL analysis; 104 randomized, 89 completed second session	Same biographical music intervention; not an independent trial	Mindfulness-based control; greater subjective distress reduction, no differential psychobiological effects	Cannot establish cortisol-specific superiority or independently replicate SOL
Bradt et al. (2024), United States	Randomized trial; 92 people with advanced cancer and chronic pain	Six weekly MT sessions; music-guided breathing, participant-selected inspirational songs, and vocal/instrumental improvisation; no fixed genre	Social attention control; pain-related self-efficacy identified as a mediator	Mediation requires causal assumptions; pain-management mechanism, not a test of legacy transmission
Ettenberger et al. (2025), Colombia	Multicentre trial; 129 randomized, 110 analysed	Single group session; participant-selected boleros, gospel, vallenatos, salsa and Hispanic ballads within a structured session	Standard care; lower post-session anxiety and better psychological/general wellbeing	Complete-data analysis; immediate outcomes; chemotherapy rather than specifically terminal care; no genre-effect comparison

Studies are ordered chronologically. These are design categories, not formal quality grades. SOL publications describe the same trial. NR-A means that genre details were not available in the abstract or accessible report checked; it does not mean that the full paper necessarily omits them. MT, music therapy; PMR, progressive muscle relaxation; QoL, quality of life; SOL, Song of Life.

**Table 2:** Selected nonrandomized, qualitative, and mixed-methods research.

Study	Design and sample/setting	Music selection or format	Contribution to the review	Limitations and applicability
O’Callaghan (1996)	Qualitative analysis of 64 songs from 39 palliative-care patients	Patient-written lyrics; no fixed genre comparison	Describes lyrical content and personally significant concerns	Song themes are not estimates of clinical improvement
Freeman et al. (2006)	Uncontrolled review of music-vigil records; 65 dying patients	Prescriptive live harp music responsive to observed condition	Pre-post changes in agitation, wakefulness, and breathing	No comparison group; concurrent care and natural changes may explain findings
Magill (2009)	Qualitative interviews with bereaved caregivers of seven hospice patients	Pre-loss MT; genre NR-A	Caregiver accounts of remembrance, connection, and hope	Retrospective perceptions; no causal estimate of bereavement benefit
O’Callaghan et al. (2009)	Reflexive group-supervision research with four music therapists	Incomplete tangible music-therapy legacies; no standardized repertoire	Examines the meaning and handling of unfinished legacy work	Practitioner perspectives; not an intervention-effect study
Lindenfelser et al. (2012)	Exploratory mixed-methods pediatric palliative-care study; United States and Australia	Family-centred MT; genre NR-A	Valued family experiences alongside mixed parental QoL findings	Small exploratory study; not evidence specifically about neonatal heartbeat recordings
Ganzini et al. (2015)	Survey of 55 family members after a loved one’s music vigil	Bedside harp/voice vigils	Perceived comfort and relaxation, with less positive impact on pain	Family perceptions and uncontrolled design; not direct patient-reported efficacy
Preissler et al. (2016), Germany	Prospective therapy documentation; 41 patients, 166 sessions	Individualized active/receptive methods; content not predetermined	Biography was one of several recurring therapeutic topics	Clinician-recorded content; no causal test of biographical integration
Economos (2018)	Phenomenological interviews with four hospice music therapists	Clinical practice with actively dying patients; no fixed genre	Assessment, intuition, countertransference, and aesthetics	Practitioner experience, not proof of patient catharsis or symptom change
Potvin et al. (2018)	Grounded-theory study; 14 bereaved hospice caregivers	Shared music experiences; genre NR-A	Resource-oriented model of caregiver resilience and relational identity	Qualitatively derived model; proposed pathways not experimentally validated
Black et al. (2020), Canada	Qualitative MAiD study; 10 patients and seven caregiver completers	Individualized listening, songwriting, singing and playing; personally significant repertoire	Life reflection, control, communication, and aesthetic experience	Small selected sample; therapist-researcher dual role; some patients did not receive MAiD
van Dokkum et al. (2023)	NICU caregiver-perspective study, including 10 professional caregivers	Heartbeat recording combined with a family-selected Song of Kin	Reported connection, memory-making, and support for parental role	Experiential findings; NICU care not necessarily end-of-life care; no long-term comparative grief outcome
Wong et al. (2025), Singapore	Qualitative study; 16 women receiving inpatient gynae-oncology palliative care	Preferences included popular piano, popular classics, classical music, national songs, and hymns	Culturally situated accounts of meaningful musical and therapeutic experiences	Single setting; English/Mandarin eligibility; no genre comparison or efficacy estimate
Valenti et al. (2026, online 2025), United States	Mixed-methods Heartbeat Project evaluation; 30 survey responses reported in results	Personalized heartbeat keepsake with selected music	Respondents described comfort, meaning, and legacy preservation	Low response and no comparator; abstract contains inconsistent caregiver counts; no causal grief estimate
Rodgers-Melnick et al. (2026), United States	Retrospective cohort; 283 hospital admissions with hematologic/oncologic conditions	Routine MT; one, two, or at least three interventions; no genre-effect analysis	No significant exposure-by-time effect for pain or daily opioid use	No untreated comparator; residual confounding; excluded hospice encounters and in-hospital deaths

Entries are chronological. Descriptions distinguish patient reports, caregiver reports, practitioner accounts, and observed outcomes. Genre NR-A indicates that genre details were not available in the accessible material checked. Sample descriptions specify the unit of analysis where possible. MT, music therapy; MAiD, medical assistance in dying; NICU, neonatal intensive care unit; NR-A, not available in the accessible report; SOL, Song of Life. QoL, quality of life.

**Table 3:** Evidence syntheses and selected descriptive clinical sources.

Source	Design/scope	Music or intervention description	Relevant contribution	Interpretive limits
Schroeder-Sheker (1994)	Historical and clinical account of music thanatology	Prescriptive bedside music, including harp and voice	Describes the origins and practice of accompaniment for dying patients	Clinical narrative, not a controlled evaluation
Aasgaard (2001)	Four short pediatric oncology clinical histories	Patient-text songwriting and musical communication	Illustrates expression and participation within a child’s social environment	Illustrative clinical material; not four independent trials
O’Brien (2004)	Practice account involving a bone marrow transplant patient	Guided songwriting; no fixed genre	Explains collaborative songwriting in an oncology setting	Not necessarily end-of-life care; cannot establish efficacy
O’Kelly (2008)	Clinical discussion with caregiver case material	Caregiver songwriting	Illustrates an approach to carer support	Practice illustration, not comparative evidence
Heath and Lings (2012)	Clinical discussion and case vignettes	Creative songwriting at end of life and in bereavement	Explores the therapeutic process and its relationship to the song produced	Descriptive evidence; no controlled outcome estimate
McConnell et al. (2016)	Updated systematic review; three eligible trials	Palliative MT compared with standard care or other care	Possible pain benefit; no support for improved overall QoL	High risk of bias in contributing evidence
Schmid et al. (2018)	Integrative review; 12 articles	Professional MT in adult palliative care; heterogeneous methods	Integrates patient and provider perspectives	Heterogeneous designs and outcomes; not a legacy-specific effect estimate
Bradt et al. (2021)	Cochrane cancer review; 81 trials, 5,576 participants	38 MT and 43 music-medicine trials; varied musical content	Possible benefits for selected psychological and physical outcomes	Many outcomes low/very low certainty; not restricted to terminal or legacy care
Giordano et al. (2022)	Commentary on pediatric end-of-life songwriting	Songwriting with children	Clinical context for pediatric expression and legacy work	Commentary, not a controlled trial or confirmed outcome cohort
Gillespie et al. (2024)	Mixed-methods systematic review; 34 caregiver studies	Varied MT approaches before and after bereavement	Qualitative accounts supportive; quantitative effectiveness equivocal	Many studies concerned dementia; limited bereavement-phase evidence and small quantitative samples

These sources provide synthesis or clinical context rather than additional independent intervention cohorts. Primary studies can overlap with Tables 1 and 2. A review’s publication type does not establish high certainty of treatment effects. MT, music therapy; QoL, quality of life.

### 3.1 The song of life trial and other randomized evidence

SOL is a three-session biographical music therapy protocol involving a personally meaningful song, reflection on its autobiographical significance, and a therapist-recorded version (Warth et al., 2019). In two German palliative-care units, 104 patients were randomized equally to SOL or relaxation exercises. The primary psychological quality-of-life outcome and global quality of life did not differ significantly between groups. Spiritual wellbeing and ego-integrity favoured SOL, with higher ratings of treatment meaningfulness and importance (Warth et al., 2021). This pattern supports a focused psycho-spiritual interpretation rather than improvement across all domains of wellbeing. Inability to blind participants, short follow-up, and recruitment from two German-language settings constrain interpretation and transferability.

Koehler et al.’s (2022) linked analysis must not be counted as an independent trial. It found a greater reduction in subjective distress with music therapy, but no significant differential psychobiological treatment effects. Cortisol decreased over time across both interventions; the study does not establish a cortisol-specific advantage of biographical music therapy or prove a biological mechanism for legacy work.

Other randomized studies address different questions. Warth et al. (2015) randomized 84 palliative-care inpatients to two sessions of live music-based relaxation or a recorded verbal relaxation exercise. Music therapy improved relaxation and wellbeing, but the between-group difference in acute pain was not significant. Gutgsell et al.’s (2013) trial in 200 palliative-care inpatients found greater immediate pain reduction after a single music-therapy session than with standard care alone. Differences in intervention content and comparators matter: a benefit against usual care does not establish the same incremental benefit against an active relaxation intervention.

Choi’s small four-arm trial concerned 32 family caregivers of hospice patients. It compared music, progressive muscle relaxation, their combination, and control. There was no significant main effect of group, so improvements over time should not be attributed specifically to music (Choi, 2010). In Colombia, Ettenberger et al. (2025) randomized 129 chemotherapy patients, with 110 included in the complete-data analysis, to a single live group music-therapy session plus standard care or standard care alone. Post-intervention state anxiety was lower, and psychological and general wellbeing were higher, in the music-therapy group. This extends the geographical range of oncology evidence but does not establish effects on legacy, bereavement, or terminal care.

More recent mechanistic work also requires outcome-specific interpretation. In a trial of 92 people with advanced cancer and chronic pain, Bradt et al. (2024) compared six weekly individual music-therapy sessions with social attention control. Mediation analyses implicated pain-related self-efficacy, rather than anxiety, mood, or emotional support, in pain outcomes. This suggests a candidate process in pain management, not confirmation of the biographical processes proposed below. Collectively, these trials support examining intervention goals and comparators explicitly rather than treating all music therapy as a uniform exposure.

### 3.2 Proposed processes of legacy and meaning-making

Three interrelated processes help organize the literature: biographical reflection, legacy creation and transmission, and emotional expression. They are informed principally by clinical accounts and qualitative research. They are not established mediators, need not occur together, and may overlap with processes in other psychosocial interventions (Schmid et al., 2018; Heath and Lings, 2012; Potvin et al., 2018).

#### 3.2.1 Biographical integration and Ego-integrity

Biographical integration refers here to reviewing and finding meaning in one’s life narrative. Ego-integrity provides a related conceptual perspective on coherence and acceptance in later life (Erikson, 1982). Personally meaningful music can provide an entry point to memories and conversation, but the availability of a musical association does not itself demonstrate therapeutic integration (Koelsch, 2014; Warth et al., 2019).

Preissler et al.’s (2016) analysis of therapy documentation from 41 terminally ill cancer patients identified biography among several recurring topics, alongside illness, coping, emotions, and social relationships. These findings describe what patients and therapists discussed; they do not demonstrate that music restored identity or that biographical work explains measured treatment effects. Receptive listening may also prompt meaningful reflection. The distinction between therapist-supported biographical work and music listening concerns the structure and therapeutic context, not an assertion that autobiographical reflection is impossible without a therapist (Schmid et al., 2018).

#### 3.2.2 Legacy creation and transmission

Legacy work involves creating or sharing something that patients and families consider meaningful, including songs, spoken messages, or recordings. O′Callaghan’s analysis of patient-written lyrics and Heath and Lings’ clinical accounts illustrate how songwriting can express relationships and personal concerns (O’Callaghan, 1996; Heath and Lings, 2012). These sources support the relevance of the activity to some patients, rather than showing that all dying people seek a tangible legacy.

Valenti et al.’s (2026) Heartbeat Project evaluation describes personalized recordings combining a patient’s heartbeat and music. Responding caregivers generally valued the keepsakes, but the limited survey response and absence of a comparison group restrict conclusions about bereavement benefit. In a qualitative study involving 14 bereaved hospice caregivers, Potvin et al. (2018) developed a resource-oriented model emphasizing the caregiving relationship and pre-illness identities. The model is grounded in caregiver accounts, but its proposed causal sequence has not been established experimentally.

#### 3.2.3 Emotional expression and music-inspired weeping

Music may offer opportunities to express grief, love, gratitude, or ambivalence without requiring all experiences to be verbalized. Magill’s interviews with bereaved caregivers described remembrance, connection, and hope associated with pre-loss music therapy (Magill, 2009). Black et al. (2020) also reported immediate emotional responses during music therapy surrounding MAiD. Such responses can be meaningful, but tears are not, by themselves, proof of catharsis or clinical improvement.

Economos interviewed four experienced hospice music therapists about work with actively dying patients. The resulting themes concerned assessment, intuitive processes, countertransference, and aesthetics (Economos, 2018). This is evidence about practitioners’ experiences, not direct evidence that emotional release improves patient outcomes. Clinical interpretation should therefore remain attentive to a person’s response, including fatigue, distress, or a wish to stop, rather than presuming that stronger emotion indicates greater benefit.

### 3.3 Songwriting as clinical practice

Songwriting is a documented form of legacy-oriented practice across adult, pediatric, and caregiver contexts. It can involve original composition, collaborative lyric writing, or adaptation of familiar material, with the amount of patient participation determined by preference and clinical circumstances (Heath and Lings, 2012; O’Brien, 2004; O’Kelly, 2008). The process may be valuable even when no polished or completed recording results.

O′Brien’s account concerns guided songwriting with a bone marrow transplant patient; this practice account illustrates an application rather than providing a controlled evaluation of end-of-life care (O’Brien, 2004). Heath and Lings describe clinical songwriting at the end of life and in bereavement, while O’Kelly illustrates a caregiver application through case material (Heath and Lings, 2012; O’Kelly, 2008). Pediatric clinical accounts also describe music-making as an opportunity for expression and connection (Aasgaard, 2001; Giordano et al., 2022). These reports illuminate practice but cannot establish comparative efficacy or identify a universally effective songwriting method.

Not all patients want to create a legacy artifact, and declining or discontinuing the activity should not be construed as a therapeutic failure. O’Callaghan et al.’s (2009) reflexive group-supervision research specifically addresses unfinished musical legacies. Their work supports attention to the meanings of incompletion and to therapists’ responses, rather than an expectation that every intervention culminate in a finished song.

### 3.4 Music thanatology: a distinct tradition

Music thanatology represents a distinct bedside practice associated with Therese Schroeder-Sheker’s work. Practitioners use live music, commonly harp and voice, adjusted in relation to the dying person’s observed condition (Schroeder-Sheker, 1994; Freeman et al., 2006). Instrumentation and responsive delivery should not be confused with a musical genre. Its immediate goals of accompaniment and comfort differ from explicit biographical reflection or legacy recording.

Freeman et al. (2006) examined records of 65 patients receiving music vigils and reported changes in observed agitation, wakefulness, and breathing following the intervention. The uncontrolled pre-post design cannot separate effects of music from concurrent care or changes in the dying process. Ganzini et al. (2015) surveyed 55 family members, who perceived benefits particularly for comfort and relaxation, with less positive impact on pain. These findings support perceived acceptability and comfort, not a claim that music reliably produces a peaceful death. Qualitative and observational evidence deserves consideration on its own terms while the absence of controlled evidence limits causal conclusions.

### 3.5 Pediatric and neonatal legacy work

Pediatric applications share an interest in expression and connection but must account for developmental stage, family involvement, and the child’s capacity and wishes. Aasgaard’s (2001) clinical account and Giordano et al.’s (2022) commentary describe pediatric music-making and songwriting, without providing randomized evidence of legacy outcomes. The direction of legacy work is not necessarily from family to child: it may involve the child’s own expression, joint creation, or family-led remembrance.

Lindenfelser et al.’s (2012) exploratory mixed-methods study concerned family-centred pediatric palliative music therapy in the United States and Australia. Parents described valued shared experiences, but quantitative quality-of-life findings were mixed.

Heartbeat-based neonatal work is better supported by van Dokkum et al.’s (2023) HeartSong study, which examined caregiver perspectives on combining an infant’s heartbeat with a family-selected Song of Kin. Reported themes included connection, memory-making, and support for the parental role. These are preliminary experiential findings from neonatal intensive care, not evidence that all participants received end-of-life care or that the recordings prevent prolonged grief. Adult and pediatric palliative heartbeat keepsakes are also described by Valenti et al. (2026), with similar limitations regarding selection and self-reported outcomes.

### 3.6 Music therapy and medical assistance in dying

Black et al.’s (2020) peer-reviewed qualitative study of music therapy surrounding MAiD included 10 patients and seven caregiver completers, together with the therapist-researcher’s observations. Patient themes included life reflection, control, communication, and aesthetic experience; some participants ultimately died without receiving MAiD. These findings support further study of patient-led musical support in this context, but do not establish comparative effectiveness or a uniquely advantageous setting for legacy work. The small selected sample and therapist-researcher dual role limit transferability.

### 3.7 Musical genre, culture, and equity

The reviewed interventions vary in both musical content and delivery. SOL uses a personally significant song, Warth et al. (2019), whereas Warth et al.’s (2015) relaxation intervention uses live monochord and voice. Wong et al.’s (2025) qualitative study in Singapore involved 16 women receiving gynae-oncology palliative care. Reported preferences included popular piano, popular classics, classical music, national songs, and hymns. Ettenberger et al. (2025) reported boleros, gospel, vallenatos, salsa, and Hispanic ballads selected around participants’ preferences in chemotherapy groups. These examples show diversity of musical material, but do not constitute controlled comparisons of genre-specific effectiveness.

The available evidence does not establish that one genre is superior for legacy or existential outcomes in palliative care. Familiarity, lyrical meaning, language, autobiographical association, religious significance, delivery, and the therapeutic relationship may all influence experience. Because these characteristics frequently occur together, observed responses should not be attributed to genre alone. Future reports should describe the selection process, patient choice, musical examples or genre where appropriate, live versus recorded delivery, and relevant cultural context (Wong et al., 2025; Warth et al., 2019; Black et al., 2020).

The literature’s concentration in North America, Europe, and Australia limits transferability, but evidence outside those settings is not absent (Gillespie et al., 2024; Wong et al., 2025). The Singapore study offers directly relevant Asian palliative-care evidence; the Colombian chemotherapy trial provides additional geographical context without answering the same legacy-focused question (Wong et al., 2025; Ettenberger et al., 2025). Neither setting represents all Asian or non-Western populations. Ethnicity, language, religion, migration history, and individual musical identity should not be inferred from the country where a study was conducted.

Indigenous populations likewise should not be treated as culturally homogeneous. Kelly et al.’s (2009) interviews with bereaved First Nations family members in northern Ontario emphasized extended-family participation, respectful communication, and appropriate care environments. Castleden et al. (2010) examined providers’ perceptions of access to Aboriginal palliative care in rural British Columbia. These are contextual palliative-care studies, not evaluations of music therapy or proof of particular musical preferences. Their implications concern asking about local needs and working with communities rather than imposing assumptions about individual or collective legacy.

Further work should include Indigenous communities beyond Canada and underrepresented Asian, African, Latin American, and other populations, with locally relevant questions and collaboration. This English-language narrative review cannot establish that no relevant studies exist in those settings. Consent to record or share music, the wishes of patients and families, and the handling of culturally significant material require attention in designing such research. Cultural adaptation should be evaluated rather than assumed to preserve the effects of an existing protocol.

### 3.8 Reporting quality and evidence gaps

The evidence supports different kinds of inference. Randomized trials can estimate comparative effects for their specific outcomes; qualitative studies explain experiences and perceived value; clinical accounts develop practice concepts. These contributions should not be collapsed into a hierarchy that equates qualitative methods with poor quality. Conversely, confidence in an evidence synthesis depends on the underlying studies. The 2021 Cochrane cancer review included 81 trials but judged much of the outcome evidence low or very low certainty (Bradt et al., 2021).

Outcome specificity is important. SOL used validated spiritual-wellbeing and ego-integrity measures, but its primary psychological quality-of-life outcome was not significantly different between groups (Warth et al., 2021). Legacy satisfaction, family connection, symptom intensity, and bereavement adjustment are not interchangeable outcomes. More consistent reporting would improve comparability, but disagreement across domains should not automatically be treated as measurement failure.

Long-term family outcomes remain insufficiently established. Gillespie et al.’s (2024) synthesis of 34 studies found encouraging qualitative accounts but equivocal quantitative findings and limited evidence for the bereavement phase. Heartbeat evaluations contribute caregiver perspectives without establishing lasting improvement in grief trajectories (Valenti et al., 2026; van Dokkum et al., 2023). Attrition, nonresponse, and the absence of suitable comparators warrant explicit attention.

Other gaps include limited genre comparisons, incomplete reporting of music selection, underrepresentation of diverse settings, and difficulty separating biographical content from attention, musical enjoyment, or therapist contact (Warth et al., 2019; Wong et al., 2025; Schmid et al., 2018). Future studies should match design and outcomes to the intervention’s purpose, report unwanted responses and nonparticipation, and distinguish acceptability from effectiveness.

### 3.9 Pain and analgesic medication use

Symptom-management evidence provides relevant context but is not evidence of a legacy-specific effect. Immediate pain reduction, sustained pain improvement, and reduced analgesic use are distinct endpoints. Gutgsell et al. (2013) found a short-term pain benefit, whereas Warth et al. (2015) did not find a significant between-group acute-pain difference against an active relaxation comparator.

In Bates et al.’s (2017) randomized transplantation study of 82 patients, the music-therapy group used fewer morphine-equivalent analgesics than controls over the specified peri-transplant period, despite similar pain scores. Median use was 24 mg versus 73 mg (*p* = 0.038). This is setting-specific oncology evidence and cannot be assumed to apply to dying patients or to biographical interventions.

Rodgers-Melnick et al.’s (2026) retrospective study examined 283 hospital admissions involving patients with hematologic or oncologic conditions who received music therapy. Comparing one, two, or at least three interventions, it found no significant exposure-by-time effect for pain intensity (*p* = 0.131) or daily opioid utilization (*p* = 0.118). Patients discharged to hospice, seen by a hospice professional during admission, or recorded as deceased at discharge were excluded (Rodgers-Melnick et al., 2026). The study therefore does not support a claim of reduced opioid use in end-of-life care. It also lacks a no-music-therapy group. Together, these findings support cautious, outcome-specific evaluation of music therapy alongside usual analgesic care.

## 4 Discussion

This review brings together evidence relevant to legacy, biographical reflection, and meaning-making through music therapy. The organizing themes of life reflection, legacy transmission, and emotional expression help compare clinical approaches but remain an interpretive synthesis. Similar experiences across studies do not establish a shared causal mechanism (Heath and Lings, 2012; Potvin et al., 2018; Schmid et al., 2018).

SOL provides directly relevant randomized evidence for a defined biographical protocol, while other trials examine relaxation, pain, anxiety, or caregiver outcomes (Warth et al., 2021; Warth et al., 2015; Gutgsell et al., 2013; Choi, 2010; Ettenberger et al., 2025). Their results should be interpreted in relation to the chosen comparator, treatment duration, population, and outcome. In particular, improved spiritual wellbeing should not be generalized to global quality of life, and short-term pain relief should not be equated with reduced medication requirements or lasting benefit (Warth et al., 2021; Bates et al., 2017; Rodgers-Melnick et al., 2026). The absence of differential cortisol effects in the SOL substudy further limits claims of a demonstrated biological pathway (Koehler et al., 2022).

The qualitative literature contributes accounts of connection, remembrance, and meaning that symptom scales may not fully capture (Magill, 2009; Potvin et al., 2018; Wong et al., 2025). These reports are clinically informative without establishing that every patient benefits or that a particular genre is necessary. Songwriting and recordings should be offered in ways that preserve choice, including the option not to create a tangible legacy. Work on incomplete legacies also cautions against valuing the finished product above the encounter (O’Callaghan et al., 2009; Heath and Lings, 2012).

Music thanatology, neonatal heartbeat recordings, and music therapy surrounding MAiD broaden understanding of accompaniment and remembrance, but they remain distinct practices with different populations and goals (Freeman et al., 2006; van Dokkum et al., 2023; Black et al., 2020). Keeping these boundaries explicit avoids applying evidence from one context to another. Similarly, wider caregiver syntheses include conditions beyond terminal cancer and should not be presented as direct confirmation of bereavement benefit from legacy recordings (Gillespie et al., 2024).

Cultural responsiveness is integral to clinical relevance, but broad labels such as Western, Asian, or Indigenous do not predict a person’s musical preferences. Locally grounded research and clear reporting of language, repertoire selection, family involvement, and context are needed before transferring intervention models across settings (Wong et al., 2025; Kelly et al., 2009; Castleden et al., 2010). The evidence currently supports individualized inquiry more strongly than prescriptions for a particular genre.

### 4.1 Positionality and limitations

The author has volunteered as a live musician at the University of Alberta Hospital in Edmonton, Alberta, Canada, through the Artists on the Wards program for 6 years. This experience includes performance in palliative-care settings and provides contextual familiarity, but may also create a bias toward positive interpretations of music’s value. Volunteer performance is distinct from professional music therapy. No personal observations are treated as research evidence in this review.

This is a single-author narrative synthesis with an English-language restriction, selective coverage, no independently duplicated screening, and no formal study-level risk-of-bias assessment. The targeted revision searches do not constitute a systematic update. Detailed genre information was not available for every report; where only an abstract or bibliographic record could be checked, the tables avoid inferring unreported repertoire or sample characteristics. These limitations restrict claims of completeness and comparative certainty. Linked trial publications and overlap between reviews and primary studies also mean that the number of reports is not the number of independent bodies of evidence.

## 5 Conclusion

Legacy-oriented and biographical music therapy may complement palliative care by offering opportunities for reflection, expression, and connection. The SOL trial provides short-term evidence for selected psycho-spiritual outcomes, while qualitative and clinical literature describes experiences that patients, families, and practitioners consider meaningful. The findings do not establish a universal legacy mechanism, superiority of one musical genre, reliable opioid reduction, or sustained bereavement benefit across populations.

Future research should prioritize independent trials of clearly described biographical interventions, careful comparator selection, longitudinal family outcomes, and culturally grounded collaboration. Reporting should distinguish genre from delivery and personal significance, and should identify acceptability, symptom change, and legacy-related outcomes separately. Clinical use should remain responsive to patients’ preferences and goals within multidisciplinary care.

## References

[ref1] Aasgaard T. (2001). An ecology of love: aspects of music therapy in the pediatric oncology environment. J. Palliat. Care 17, 177–181. doi: 10.1177/082585970101700310, 11816759

[ref2] Baethge C. Goldbeck-Wood S. Mertens S. (2019). SANRA-a scale for the quality assessment of narrative review articles. Res Integr Peer Rev. 4:5. doi: 10.1186/s41073-019-0064-8, 30962953 PMC6434870

[ref3] Bates D. Bolwell B. Majhail N. S. Rybicki L. Yurch M. Abounader D. . (2017). Music therapy for symptom management after autologous stem cell transplantation: results from a randomized study. Biol. Blood Marrow Transplant. 23, 1567–1572. doi: 10.1016/j.bbmt.2017.05.015, 28533058

[ref4] Black S. Bartel L. Rodin G. (2020). Exit music: the experience of music therapy within medical assistance in dying. Healthcare (Basel) 8:E331. doi: 10.3390/healthcare8030331, 32927626 PMC7551927

[ref5] Bradt J. Dileo C. Myers-Coffman K. Biondo J. (2021). Music interventions for improving psychological and physical outcomes in people with cancer. Cochrane Database Syst. Rev. 2022:CD006911. doi: 10.1002/14651858.cd006911.pub4, 34637527 PMC8510511

[ref6] Bradt J. Leader A. Worster B. Myers-Coffman K. Bryl K. Biondo J. . (2024). Music therapy for pain management for people with advanced cancer: a randomized controlled trial. Psychooncology 33:e70005. doi: 10.1002/pon.70005, 39450934 PMC11778920

[ref7] Breitbart W. Rosenfeld B. Gibson C. Pessin H. Poppito S. Nelson C. . (2010). Meaning-centered group psychotherapy for patients with advanced cancer: a pilot randomized controlled trial. Psychooncology 19, 21–28. doi: 10.1002/pon.1556, 19274623 PMC3648880

[ref8] Castleden H. Crooks V. A. Hanlon N. Schuurman N. (2010). Providers’ perceptions of aboriginal palliative care in British Columbia’s rural interior. Health Soc. Care Community 18, 483–491. doi: 10.1111/j.1365-2524.2010.00922.x, 20500225

[ref9] Choi Y. K. (2010). The effect of music and progressive muscle relaxation on anxiety, fatigue, and quality of life in family caregivers of hospice patients. J. Music. Ther. 47, 53–69. doi: 10.1093/jmt/47.1.53, 20635523

[ref10] Economos A. D. (2018). Music therapy when death is imminent: a phenomenological inquiry. J. Music. Ther. 55, 309–339. doi: 10.1093/jmt/thy011, 29992294

[ref11] Erikson E. H. (1982). The Life Cycle Completed. New York: W. W. Norton.

[ref12] Ettenberger M. Reyes-Aragón L. Díaz A. M. Suárez R. Amarillo M. Naranjo M. . (2025). Effect of group music therapy on state-anxiety and wellbeing levels of oncology patients undergoing chemotherapy: a multi-center randomized clinical trial. Front. Psych. 16:1658503. doi: 10.3389/fpsyt.2025.1658503, 40980027 PMC12447645

[ref13] Freeman L. Caserta M. Lund D. Rossa S. Dowdy A. Partenheimer A. (2006). Music thanatology: prescriptive harp music as palliative care for the dying patient. Am. J. Hosp. Palliat. Care 23, 100–104. doi: 10.1177/104990910602300206, 16572747

[ref14] Ganzini L. Rakoski A. Cohn S. Mularski R. A. (2015). Family members’ views on the benefits of harp music vigils for terminally-ill or dying loved ones. Palliat. Support. Care 13, 41–44. doi: 10.1017/s1478951513000564 24128665

[ref15] Gillespie K. McConnell T. Roulston A. Potvin N. Ghiglieri C. Gadde I. . (2024). Music therapy for supporting informal carers of adults with life-threatening illness pre- and post-bereavement; a mixed-methods systematic review. BMC Palliat. Care 23:55. doi: 10.1186/s12904-024-01364-z, 38408966 PMC10898157

[ref16] Giordano F. Rutigliano C. Baroni M. Grassi M. Muggeo P. Santoro N. (2022). Music therapy and pediatric palliative care: songwriting with children in the end-of-life. World J. Pediatr. 18, 695–699. doi: 10.1007/s12519-022-00578-6, 35773435

[ref17] Grant M. J. Booth A. (2009). A typology of reviews: an analysis of 14 review types and associated methodologies. Health Inf. Libr. J. 26, 91–108. doi: 10.1111/j.1471-1842.2009.00848.x, 19490148

[ref18] Gutgsell K. J. Schluchter M. Margevicius S. DeGolia P. A. McLaughlin B. Harris M. . (2013). Music therapy reduces pain in palliative care patients: a randomized controlled trial. J. Pain Symptom Manag. 45, 822–831. doi: 10.1016/j.jpainsymman.2012.05.008 23017609

[ref19] Heath B. Lings J. (2012). Creative songwriting in therapy at the end of life and in bereavement. Mortality 17, 106–118. doi: 10.1080/13576275.2012.673381

[ref20] Kelly L. Linkewich B. Cromarty H. St Pierre-Hansen N. Antone I. Giles C. (2009). Palliative care of first nations people: a qualitative study of bereaved family members. Can. Fam. Physician 55, 394–395. PMID 19366951. PMC2669014 19366951

[ref21] Koehler F. Kessler J. Stoffel M. Weber M. Bardenheuer H. J. Ditzen B. . (2022). Psychoneuroendocrinological effects of music therapy versus mindfulness in palliative care: results from the ‘song of life’ randomized controlled trial. Support Care Cancer 30, 625–634. doi: 10.1007/s00520-021-06435-y, 34355279 PMC8636432

[ref22] Koelsch S. (2014). Brain correlates of music-evoked emotions. Nat. Rev. Neurosci. 15, 170–180. doi: 10.1038/nrn3666, 24552785

[ref23] Lindenfelser K. J. Hense C. McFerran K. (2012). Music therapy in pediatric palliative care: family-centered care to enhance quality of life. Am. J. Hosp. Palliat. Care 29, 219–226. doi: 10.1177/1049909111429327, 22144660

[ref24] Magill L. (2009). The spiritual meaning of pre-loss music therapy to bereaved caregivers of advanced cancer patients. Palliat. Support. Care 7, 97–108. doi: 10.1017/s1478951509000121, 19619379

[ref25] McConnell T. Scott D. Porter S. (2016). Music therapy for end-of-life care: an updated systematic review. Palliat. Med. 30, 877–883. doi: 10.1177/0269216316635387, 26944533

[ref26] O’Brien E. (2004). The language of guided song writing with a bone marrow transplant patient. Voices 4. doi: 10.15845/voices.v4i1.144

[ref27] O’Callaghan C. C. (1996). Lyrical themes in songs written by palliative care patients. J. Music. Ther. 33, 74–92. doi: 10.1093/jmt/33.2.74

[ref28] O’Callaghan C. Petering H. Thomas A. Crappsley R. (2009). Dealing with palliative care patients’ incomplete music therapy legacies: reflexive group supervision research. J. Palliat. Care 25, 197–205. doi: 10.1177/082585970902500307, 19824281

[ref29] O’Kelly J. (2008). Saying it in song: music therapy as a carer support intervention. Int. J. Palliat. Nurs. 14, 281–286. doi: 10.12968/ijpn.2008.14.6.30023, 18928132

[ref30] Potvin N. Bradt J. Ghetti C. (2018). A theoretical model of resource-oriented music therapy with informal hospice caregivers during pre-bereavement. J. Music. Ther. 55, 27–61. doi: 10.1093/jmt/thx019, 29438566

[ref31] Preissler P. Kordovan S. Ullrich A. Bokemeyer C. Oechsle K. (2016). Favored subjects and psychosocial needs in music therapy in terminally ill cancer patients: a content analysis. BMC Palliat. Care 15:48. doi: 10.1186/s12904-016-0122-7, 27176621 PMC4866407

[ref32] Rodgers-Melnick S. N. Gunzler D. Love T. E. Koroukian S. M. Beno M. Rao S. . (2026). Longitudinal effects of inpatient music therapy dosage on pain intensity and opioid utilization among patients with hematologic or oncologic conditions: a retrospective cohort study. Support Care Cancer 34:620. doi: 10.1007/s00520-026-10865-x, 42249214 PMC13241398

[ref33] Saunders C. (2000). The evolution of palliative care. Patient Educ. Couns. 41, 7–13. doi: 10.1016/s0738-3991(00)00110-5, 10900362

[ref34] Schmid W. Rosland J. H. von Hofacker S. Hunskår I. Bruvik F. (2018). Patient’s and health care provider’s perspectives on music therapy in palliative care—an integrative review. BMC Palliat. Care 17:32. doi: 10.1186/s12904-018-0286-4, 29463240 PMC5819707

[ref35] Schroeder-Sheker T. (1994). Music for the dying: a personal account of the new field of music-thanatology—history, theories, and clinical narratives. J. Holist. Nurs. 12, 83–99. doi: 10.1177/089801019401200113, 7806853

[ref36] Valenti K. Cleveland M. Hamrick K. Vickers J. Stockdill M. L. Hicks J. . (2026). My heart will go on: caregiver experiences of a legacy intervention in acute palliative care. J. Pain Symptom Manag. 71, 290–299. doi: 10.1016/j.jpainsymman.2025.10.019, 41167573

[ref37] van Dokkum N. H. Fagan L. J. Cullen M. Loewy J. V. (2023). Assessing HeartSong as a neonatal music therapy intervention: a qualitative study on personal and professional caregivers’ perspectives. Adv. Neonatal Care 23, 264–271. doi: 10.1097/anc.0000000000001068, 37075326

[ref38] Warth M. Keßler J. Hillecke T. K. Bardenheuer H. J. (2015). Music therapy in palliative care. Dtsch. Arztebl. Int. 112, 788–794. doi: 10.3238/arztebl.2015.0788, 26806566 PMC4671329

[ref39] Warth M. Koehler F. Brehmen M. Weber M. Bardenheuer H. J. Ditzen B. . (2021). "Song of life": results of a multicenter randomized trial on the effects of biographical music therapy in palliative care. Palliat. Med. 35, 1126–1136. doi: 10.1177/02692163211010394, 33876660 PMC8188998

[ref40] Warth M. Koehler F. Weber M. Bardenheuer H. J. Ditzen B. Kessler J. (2019). "Song of life (SOL)" study protocol: a multicenter, randomized trial on the emotional, spiritual, and psychobiological effects of music therapy in palliative care. BMC Palliat. Care 18:14. doi: 10.1186/s12904-019-0397-6, 30700278 PMC6354383

[ref41] Wong K. C. Magee W. L. Mun S. Sim Y. Z. Nair T. Tewani K. (2025). The value of inpatient music therapy for gynae-oncology palliative care in Singapore. Front. Psych. 16:1577545. doi: 10.3389/fpsyt.2025.1577545, 40496822 PMC12150798

